# Analytical applications of MIPs in diagnostic assays: future perspectives

**DOI:** 10.1007/s00216-015-9137-9

**Published:** 2015-11-21

**Authors:** Thomas S. Bedwell, Michael J. Whitcombe

**Affiliations:** Department of Chemistry, College of Science and Engineering, University of Leicester, Leicester, LE1 7RH UK

**Keywords:** Molecularly imprinted polymers, Molecularly imprinted sorbent assay, Immunoassay, Radioassay, Fluoroimmunoassay, BELISA

## Abstract

Many efforts have been made to produce artificial materials with biomimetic properties for applications in binding assays. Among these efforts, the technique of molecular imprinting has received much attention because of the high selectivity obtainable for molecules of interest, robustness of the produced polymers, simple and short synthesis, and excellent cost efficiency. In this review, progress in the field of molecularly imprinted sorbent assays is discussed—with a focus on work conducted from 2005 to date.

**Graphical Abstract:**
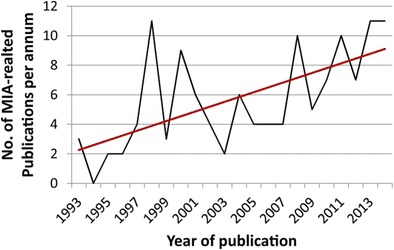
A growing trend in recent years has been the use of molecularly imprinted polymers as replacements for antibodies in various assay formats, as indicated by a steady increase in publications in the area (see graph)

A growing trend in recent years has been the use of molecularly imprinted polymers as replacements for antibodies in various assay formats, as indicated by a steady increase in publications in the area (see graph)

## Introduction

Specific receptor–ligand interactions are a fundamental process in biological systems, essential for the generation of physiological responses to substances such as hormones, proteins, cellular markers, antigens etc. The specific nature of biological recognition, in particular of antibodies and enzymes, has led to their exploitation as the recognition element of choice in many assay systems and biosensors. However, despite possessing high specificity and sensitivity for their respective ligands, biomolecules suffer the disadvantages of fragility and high cost. The ability to mimic the highly specific nature of antibodies and enzymes in more robust and lower cost materials has been of great interest to researchers in the field. Consequently, much effort has been expended in the design and synthesis of artificial materials with biomimetic properties. Among these, the technique of molecular imprinting has received much attention because of the high selectivity obtainable for molecules of interest. Coupled with the advantages of short synthesis time, robustness, regeneration (and consequently cost efficiency), as well as cheap initial production, molecularly imprinted polymers (MIPs) provide an attractive alternative to conventional biological receptors.

**Fig. 1:**
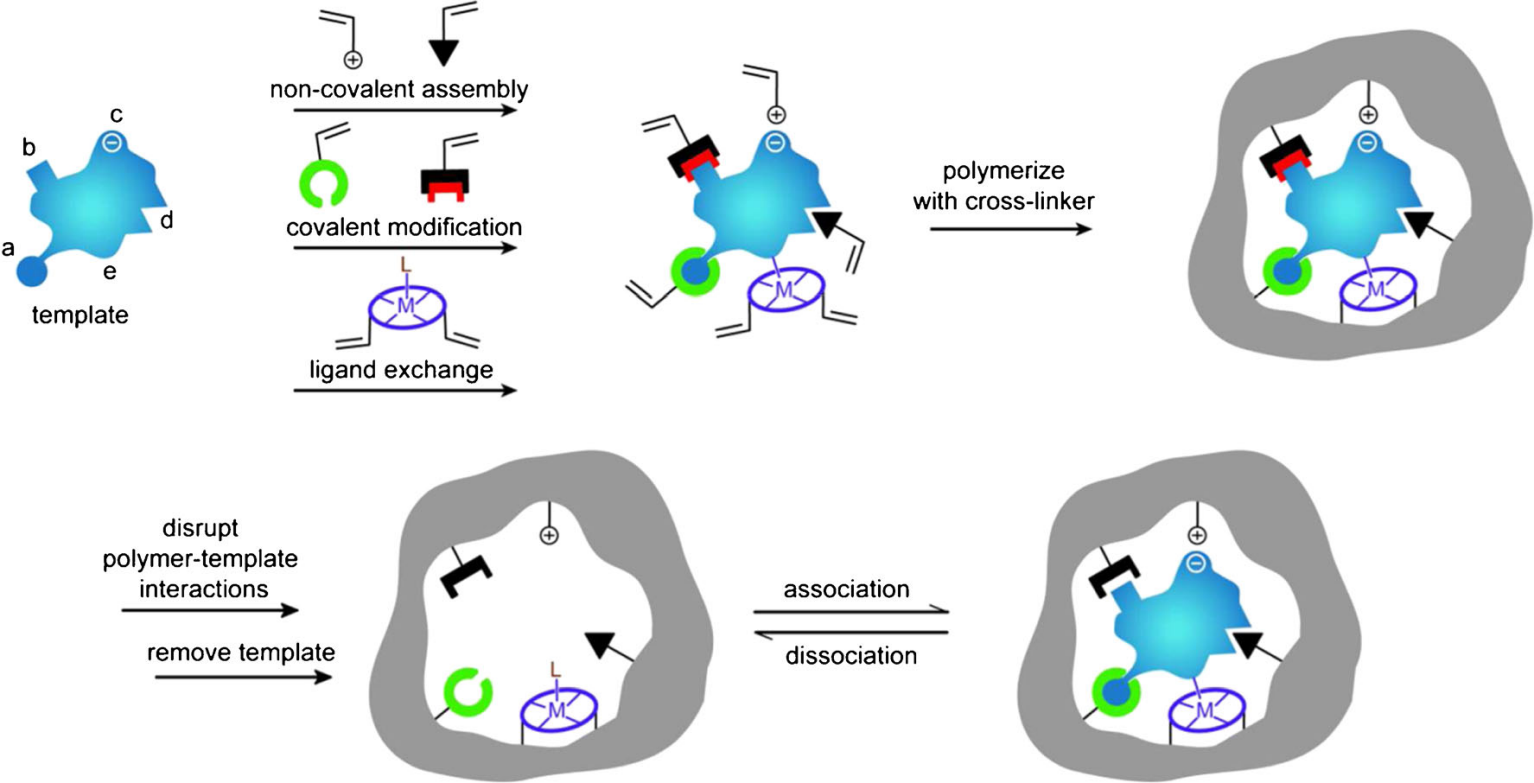
Schematic representation of the molecular imprinting process [1]. Reproduced with permission from: Molecular Imprinting Science and Technology: a survey of the literature for the years up to and including 2003, Alexander C et al. Journal of Molecular Recognition, Vol. 19:2, Copyright © 2003, John Wiley and Sons

The process of molecular imprinting involves the synthesis of a polymeric material in the presence of a template, producing complementary recognition sites in the imprinted polymer that are specific for the template molecule (Fig. 1). This is achieved by addition of the template to a polymerization mixture comprising functional monomer, cross-linking agent, and solvent (sometimes referred to as the porogen). A prepolymerization complex is initially formed, with functional monomers arranging themselves around the template in a manner influenced by the shape and chemical properties of the template. Subsequent polymerization of this complex fixes the monomers in this arrangement, and removal of the template affords a complementary recognition site for the template molecule. In this way, an imprinted polymer is constructed with molecular memory for the substrate of interest by a self-assembly process [1–6].

## The development of molecularly imprinted sorbent assays, a brief history

Once imprinted polymers could be generated with affinity and selectivity comparable to biological antibodies, the potential to compete as a genuine synthetic alternative in assays became possible. In 1993, Vlatakis et al. described such an assay, coining the term “molecularly imprinted [sorbet] assay” (MIA) [7]. Imprinted polymers of ethylene dimethacrylate-co-methacrylic acid were prepared by bulk polymerization against two chemically unrelated drugs, theophylline (a bronchodilator) and diazepam (a tranquilizer). The MIPs were successfully employed in assays analogous to competitive radiolabeled immunoassays, achieving impressive results: for theophylline, measurements were linear over the range of 14–224 μM, the results of analysis of serum samples from 32 patients showed excellent correlation with those obtained using the enzyme-multiplied immunoassay technique (EMIT), and cross-reactivity against other major metabolites and structurally similar compounds was shown to be similar to that observed with biological antibodies. Whilst these results were encouraging, the MIA method was more cumbersome than EMIT as a consequence of the necessary extraction of analyte from the biological sample prior to analysis, due to the polymers giving optimal binding and selectivity only in organic solvents.

Molecular imprinting of morphine and the endogenous neuropeptide [Leu5]-enkephalin in methacrylic acid-ethylene glycol dimethacrylate copolymers and their application to a similar radioactive ligand binding assay were described by Andersson et al. in 1995 [8]. These MIPs demonstrated high binding affinity and selectivity in aqueous buffers as well as organic solvents, presenting a major breakthrough for molecular imprinting technology since the binding reactions were now occurring under conditions relevant to biological systems. Although efficient rebinding was possible in aqueous buffers, the affinities and selectivities obtained were lower than those obtained in the best organic solvents.

The influence of parameters affecting ligand binding in water were subject to further study, and an optimization of the assay conditions for (*S*)-propranolol afforded similarly high sensitivity under both organic and aqueous conditions, with limits of detection (LoD) as low as 5.5 and 6.0 nM, respectively [9]. This represented a 100- to 1000-fold improvement compared to LoDs previously achieved with MIPs, placing both aqueous and organic solvent-based MIAs on the same level as immunoassays using biological antibodies.

Having developed analyte-MIP systems that may be utilized equally well using an aqueous buffer or an organic solvent, progression into direct assay of biological samples was next to be reported. Using (*S*)-propranolol MIPs prepared in the same manner as the aforementioned study, a radiolabeled assay for direct determination of the concentration of (*S*)-propranolol in human plasma and urine was accomplished over the range 20 to 1000 nM with accuracies of 89 %-107 % and 91 %-125 %, respectively [10]. These results demonstrated that it was possible to carry out molecular imprint-based assays of biological samples without prior sample clean up.

Whilst attempting to develop a detection system for the herbicide 2,4-dichlorophenoxyacetic acid (2,4-D), Haupt et al., following limited success imprinting in the presence of nonpolar solvents, investigated whether specific noncovalent molecular imprints could be obtained in the presence of polar solvents using a combination of the hydrophobic effect and ionic interactions [11]. The template, 2,4-D functioned well in this role owing to its hydrophobic aromatic ring and ionisable carboxyl group. Polymers synthesized using 4-vinylpyridine as functional monomer and ethylene glycol dimethacrylate as cross-linker demonstrated an appreciable binding specificity and sensitivity comparable to indirect enzyme-linked immunosorbent assay (ELISA) or radioimmunoassay (RIA). These findings extended the potential applicability of noncovalent molecular imprinting to assays in cases where either the use of polar solvents may be required, or the target molecule may lack the functionality required for imprinting in nonpolar solvents.

Despite the undeniable advantage provided by the possibility of using radiolabeled tracers with identical chemical structure to the analyte of interest, issues concerning the commercial unavailability of isotopic-labeled tracers for many compounds of interest coupled with apprehensions over the handling and disposal of radionuclides made the development of assays based on other labeling and detection methods an attractive proposition. The first MIA to remove the necessity for radiolabeling was developed by Piletsky et al., who utilized competition between a fluorescein-labeled triazine analogue and unlabeled triazine for specific binding sites in an imprinted polymer to achieve an optical sensor based on fluorescence measurement [12]. This assay exhibited sensitivity for triazine over the range 0.01–100 mM, demonstrating that highly sensitive optical assays based on safe fluorescent labels could offer a promising alternative to the currently adopted radiolabeling approach.

An alternative approach to utilize changes in fluorescence as the detection mechanism led to the design of a fluorescent functional monomer: *trans*-4-[p-(*N*,*N*-dimethylamino)styryl]-*N*-vinylbenzenepyridinium chloride [13]. This monomer combined microenvironmental sensitive fluorescence, attributable to intramolecular charge-transfer behavior, with a positive charge capable of association with negatively charged nucleotides, together with a vinyl group, necessary for incorporation into the polymer matrix. With these characteristics, the monomer was incorporated within a methacrylate polymer, where it acted as both the recognition and detection element for the fluorescence determination of adenosine 3′:5′-cyclic monophosphate (cAMP) in aqueous media. The binding of cAMP led to a quantifiable quenching of fluorescence, whereas almost no effect was observed in the presence of the structurally similar molecule guanosine 3′5′-cyclic monophosphate (cGMP). Whilst this demonstrated the utility of modifying the MIP rather than analyte in order to elicit a response to binding, the use of fluorophores, which act simultaneously as both recognition element and detection element means that new monomers will need to be specifically designed for each class of analyte.

Another substitute for radiolabeling commonly employed in immunoassays involves the incorporation of enzyme labels; however, these initially seemed less suitable in MIAs for two reasons. First, enzymes often only work in aqueous buffers, and second, the hydrophobic nature and highly cross-linked structure of the polymers was proposed to limit the access of large protein molecules to the imprinted sites. As previously discussed, MIPs that perform well in aqueous solvents had been developed; however, the second problem of binding site accessibility required the development of new synthesis methods such as the preparation of monodisperse spherical imprinted polymer particles in the submicron-size range via precipitation polymerization. Ye et al. developed the synthesis of particles imprinted with theophylline and 17β-estradiol and demonstrated radioligand binding assays for the two analytes [14]. The imprinted microspheres demonstrated higher binding site densities and more rapid kinetics as a direct consequence of their small diameter. The potential use of molecularly imprinted microspheres in ELISA-like assays was tested by Surugiu et al. for the herbicide 2,4-D and using the enzyme label tobacco peroxidase as a conjugate tracer for colorimetric and chemiluminescence detection, with calibration curves obtained ranging over 40–600 μg mL^−1^ and 1–200 μg mL^−1^, respectively [15]. Even though this assay was still less sensitive than some antibody-based assays, the findings showed for the first time that imprinted polymers could be compatible with enzyme labels, broadening the potential for the application of MIPs in immunoassay-type applications. Identifying the ever-increasing demand for automated, high-throughput assaying and screening of natural products, as well as of biological and chemical combinatorial libraries, the same group decided to adapt their ELISA-type MIP-based imaging assay for this purpose [16]. Microtiter plates (96 or 384 wells) were coated with polymer microspheres imprinted with 2,4-D, which were fixed in place using poly(vinyl alcohol) as glue. Using a competitive format, the amount of polymer-bound 2,4-D-peroxidase conjugate was quantified using luminol as the chemiluminescent substrate. Light emission was consequently measured in a high-throughput imaging format with a CCD camera-based imaging system, allowing simultaneous measurement of a large number of samples. The detection limit of 2,4-D in this assay was 34 nM, with a useful range from 68 nM to 680 μM—a dynamic range only slightly narrower than that reported for antibody-based assays (although the antibody-based assays did have lower detection limits).

**Fig. 2:**
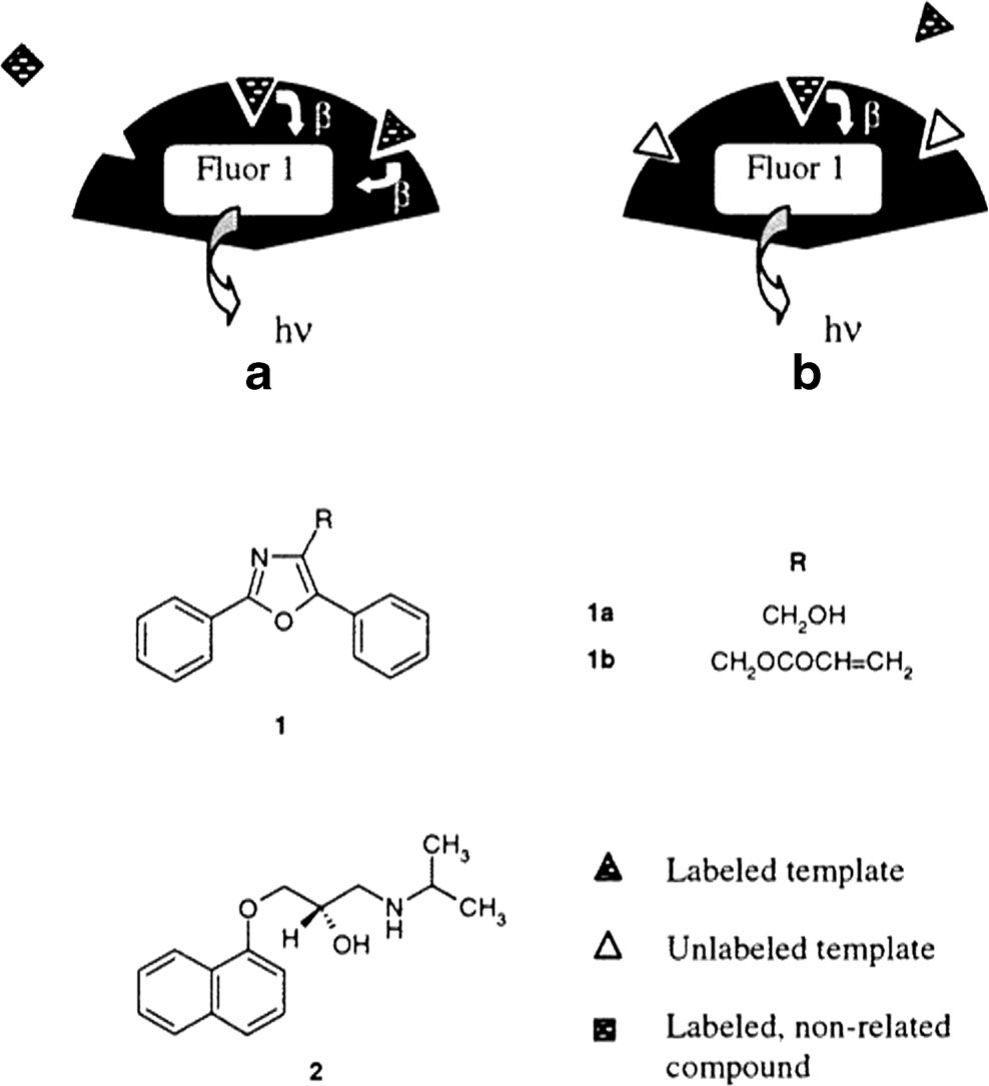
Schematic representation of chemical sensing with an imprinted polymer through proximity scintillation [18]. The polymerizable fluor **(1a)** is incorporated into imprinted particles with affinity for the template, naproxen **(2)**. The fluor emits light in response to β-decay of tritium-labeled naproxen, but not other labeled analytes **(a)**; competition between radiolabeled naproxen and free (unlabeled naproxen) **(b)** can be used to quantify the analyte without separation of the bound and free components. Reprinted with permission from Ye L, Mosbach K (2001) Journal of the American Chemical Society 123(12):2901–2902 Copyright 2001 American Chemical Society

Further optimization for high throughput screening purposes led to a novel assay aimed at eliminating the requirement for a separation step prior to quantification of the target analyte, in order to greatly increase sample throughput [17]. Generation of the binding signal was based on the principle of proximity scintillation between a scintillation fluor covalently incorporated into the MIP microparticles during preparation and the tritium-labeled analyte. Following radiolabeled ligand binding, the scintillation fluor converts incident β-radiation into a fluorescent signal, removing any need for separation of bound and unbound analyte prior to signal quantification (Fig. 2). Although this was the first demonstration of a homogenous MIP assay, the use of radiolabeled tracers was a step back from recent advances, where their usage was largely replaced by that of fluorescent and enzymatic tracers for reasons previously discussed.

## Recent developments in MIP-based assays

Of the MIAs developed since the initial work of Vlatakis et al., the majority can be classified into one of three categories determined by the type of label used for signaling: radio-labeling, fluorescence-labeling, or enzyme-linked. Recent years have seen the emergence of numerous novel assay types that do not fall into these categories; however, as each is seemingly unique in its approach, these have been grouped simply as “others” for simplicity. As the focus of this review is on recent advances, only assays reported from 2005 onwards have been included; however numerous excellent reviews covering developments made in the preceding years are available [18–27].

### Radio-labeled MIAs

A series of significant breakthroughs in MIP technology came as a result of novel synthetic methods to generate spherical, molecularly imprinted beads as an alternative to conventional MIP particles produced through bulk polymerization followed by grinding into small particles. Various approaches were developed, such as dispersion polymerization [28], suspension polymerization [29–31], activated swelling and thermal polymerization [32], precipitation polymerization [33–49], distillation precipitation polymerization [50, 51], core-shell polymerization [52–58], surface grafting methods [59–63], Pickering emulsion polymerization [64–66], hierarchical imprinting in porous silica [67, 68], and mini-emulsion polymerization [69], allowing for a diverse number of strategies for generating regular sized beads with narrow size distributions for different applications. Some of these methods have been reviewed by Pérez-Moral and Mayes [70]. Numerous investigations were thus carried out in order to assess the potential advantages of these new MIP formats in MIAs, with most being initially tested through incorporation into radio-labeled MIAs.

Based on previous work on polymerization precipitation, the group of Wei et al. reported an optimization of the technique for the preparation of 17β-estradiol imprinted nanospheres for use in radio-labeled MIAs [71]. This work focused on accurate control and optimization of the governing parameters for precipitation polymerization, taking into consideration the nature of the cross-linker, the monomer concentrations, and the polymerization temperature, and their consequent effects on the imprinted nanospheres generated. From these investigations, 17β-estradiol imprinted beads of 400 nm diameter were used in the development of a competitive binding assay, which showed a linear detection range from 0.01 to 1000 μg mL^−1^ with significant stereoselectivity for 17β-estradiol over its α-epimer.

Similar studies were performed by Ye et al., who successfully synthesized (*R*,*S*)-propranolol imprinted spherical nanoparticles of 130 nm with uniform size distribution by modifying the conditions of precipitation polymerization [72]. Through varying the composition of the cross-linker it was found that the particle size could be reasonably controlled over the range 130 nm to 2.4 μm, whilst the favorable binding properties remained intact. This led to the development of a highly enantioselective competitive radioligand binding assay, where the small MIP nanoparticles exhibited 20 times affinity for (*S*)-propranolol over the (*R*)-enantiomer, demonstrating a six- to sevenfold increase over previously reported irregular particles.

Aside from precipitation polymerization, Kempe and Kempe reported modifications on suspension polymerization in mineral oil for the preparation of (R,S)-propranolol imprinted microspheres [73]. The one-step synthesis avoided the use of water and stabilizer/surfactant, which had been a criticism of other techniques because of interference with hydrogen bonds effecting template–monomer complex formation during noncovalent imprinting. The size of beads synthesized was controllable over the range 1–100 μm, which were obtained in almost quantitative yield, with higher binding capacities observed in comparison to MIP particles prepared through bulk polymerization, likely due to better accessibility of binding sites in the spherical beads. The MIP microbeads were subsequently used for analysis of propranolol in human serum samples in a 96-well plate radio-labeled MIA, which was effective in determining propranolol concentration between 1 mM and 1 μM.

Following these optimization studies, the use of radio-labels in MIAs saw a huge decline as fluorescence- and enzyme-labeling became more popular, for reasons previously discussed. A rare example saw their use in the evaluation of a molecularly imprinted polymer for the selective recognition of testosterone [74]. Whilst previous efforts had been made to synthesize testosterone-templated polymers [75–79], these had failed to display impressive imprinting factors, the best reported being around 4, making them unsuitable for an application as an antibody mimic. This study aimed to improve on this, with the intention of optimizing testosterone imprinted MIPs in an aqueous environment for use in a radiolabeled MIA. The imprinted polymers developed showed appreciable binding affinity with association constants, *K*_*a*_ = 3.3 × 10^7^ M^−1^, whilst the nonimprinted controls bound virtually no radiolabeled testosterone, leading to a high imprinting factor compared with those previously reported. When applied to a radio assay in an aqueous environment, the molecularly imprinted polymers achieved an IC_50_ of 9 μM, making them less sensitive than commercial antibody kits; however, the selectivity exhibited was higher for the MIPs.

### Fluorescence-based MIAs

With the decline in use of radio-labeled tracers, a consequent rise in fluorescent-labeled MIAs occurred. In a typical fluorescence-labeled MIA, the target analyte is used as the template during MIP generation, whilst a fluorescent probe with similar structure is employed in competition with free analyte for binding to the polymer during the assay. This allows for sensitive and quantitative analysis through detection of the fluorescence signal. Despite its advantages, fluorescently labeled MIAs are hindered somewhat by the necessity to modify the target analyte in cases where there is no inherent fluorescence, in order to detect a signal. This is usually achieved through the addition of a fluorescent tag/group, making the structure of the probe chemically different to the analyte. The fluorescent conjugate may therefore display different binding behavior to the original analyte, which could impact on the sensitivity and selectivity of the assay. Nevertheless, impressive results have been achieved with this MIA format, with some recent developments, such as the incorporation of quantum dots, eliminating these problems entirely.

#### Heterogeneous fluorescent assays

Heterogeneous fluorescence-based assays are characterized by the physical separation of bound and unbound analyte prior to measuring the fluorescence intensity of the supernatant (or polymer) in order to perform a quantitative analysis.

Modification with pyrene or dansyl moieties led to the development of novel, highly fluorescent derivatives of the β-lactam antibiotics [80]. These compounds were ideal for optical sensing purposes and were, hence, employed in an imprinted-polymer based competitive assay for penicillin G (PenG) [81]. Selection of the most appropriate probe was conducted using radio-labeled competitive assays, with pyrenemethylacetamido penicillanic acid (PAAP) showing the most promise from the candidate library. The resulting fluorescence assay exhibited a dynamic range of 3–890 μM in 99:1 acetonitrile-water solution, with reasonable degrees of cross-reactivity (from 57 % to 0 %). When applied to the analysis of PenG in a commercial pharmaceutical formulation, recoveries from 92 % to 103 % were found. This assay was later adapted to an automated flow-injection MIA system, combining the simplicity of flow methods with the sensitivity and selectivity of the fluorescence detection [82]. The analyte and a fixed concentration of PAAP probe were injected into the MIP-packed reactor, where competition for the binding sites of the MIPs imprinted with penicillin G procaine salt occurred. Following application of a desorbing solution, the fluorescence of the labeled derivative eluted from the sorbent was measured and related to the analyte concentration in the sample. When applied to the direct analysis of PenG in spiked urine samples, mean recoveries of 92 % were observed, over a dynamic range from 787 nM to 17.1 μM. The total analysis time was 14 min per determination, with the MIP reactor capable of performing 150 cycles without significant loss of recognition. Furthermore, use of novel urea-based functional monomers in the MIP-synthesis facilitated compatibility of the system with aqueous samples—a first for automated MIAs.

Following the success of radio-labeled MIAs based on MIP micro- and nanoparticles, controlled radical polymerization was explored as a method for the synthesis of surface-imprinted core-shell nanoparticles [83]. Surface reversible addition fragmentation chain transfer (RAFT) polymerization was utilized on the surface of functionalized silica nanoparticles in the presence of 2,4-dichlorophenoxyacetic acid as template. The nanoparticles afforded by this process were subsequently applied to fluorescent-labeled MIAs using 7-carboxy-4-methylcoumarin (CMMC) as fluorescent probe. Whilst the nanoparticles generated showed no advantages over conventional irregular particles with regards to cross-reactivity, this new technology demonstrated a robust and controllable synthesis with more freedom for monomer/solvent compositions.

Generally, the preparation of MIPs uses single-template imprinting; however, reports of MIPs containing multiple sites with the ability to recognize two or even three molecules are known [84–86]. In an attempt to prepare a receptor model for biological mixed neurotransmitter receptors, Suedee et al. synthesized a dual dopamine/serotonin-selective MIP by bulk polymerization using methacrylic acid and acrylamide as functional monomers, together with *N*,*N′*-methylene bisacrylamide as cross-linker in the presence of both templates, dopamine and serotonin [87]. This dual-MIP was used in a competitive binding assay, where quantification was achieved by using the native templates as fluorescent probes. In this manner, the assay was used to attain the ligand binding activities of a series of ergot family alkaloids, in order to assess their ability to displace dopamine/serotonin from the MIP binding sites. Results were comparable to those obtained from a competitive immunoassay using receptors derived from rat hypothalamus, demonstrating binding affinities in the micromolar or submicromolar range and showing that MIPs can be capable of mimicking natural receptors in their interactions with drug targets.

#### Homogeneous fluorescent assays

In contrast to heterogeneous assays, homogeneous assays allow direct analyte measurement without the need for a physical separation step; however, this does mean that a more elaborate method for recognizing bound analyte as opposed to unbound analyte is required.

With the intention of combing the principles of a homogenous MIA and the use of a fluorescent probe, Hunt et al. developed a fluorescence polarization molecular imprinted sorbent assay for 2,4-D [88]. When the fluorescent probe, in this case 7-carboxy-4-methylcoumarin, binds to a MIP in solution, its tumbling rate falls and, consequently the measured fluorescence will be more isotropic than that of free probe, which tumbles faster. The fluorescence polarization hence increases with the percentage of probe bound, or decreases with the amount of competing analyte. In order to perform fluorescence measurements on a mixture of a fluorophore and polymer particles in solution, it was important that fluorescence could be distinguished from the scattering of excitation light by the polymer particles. This required the excitation and emission wavelengths to be well separated, and the polymer particles to be very small. Micrometer-sized particles, as previously, used were therefore too large and, consequently, the paper demonstrated for the first time that MIP microgels of diameter less than 300 nm could indeed have affinities and selectivities similar to those of bulk polymers. The limit of detection of the assay was 10 μM for 2,4-D, while selectivity was shown for the template molecule over the related herbicides 3,4-dichlorophenoxy acid and 2,4-dichlorophenoxybutyric acid.

A similar MIA utilizing fluorescence polarization as an analytical technique was also developed for the direct detection of fluoroquinolone antibiotics in food and environmental samples [89]. As the fluoroquinolones of interest display inherent fluorescence, the need to integrate an additional probe into the system was not necessary, unlike in the previous example. Water-compatible MIP nanoparticles were synthesized with enrofloxacin (ENRO) as the imprinting template; however, this also showed similar affinity towards ciprofloxacin and norfloxacin. The assay was successfully applied to determine fluoroquinolones in real samples without any prior concentration step by simply adding a known amount of MIP, with no interference from sample components observed. In tap water, the limit of detection for ENRO was 0.1 nM using 5 μg mL^−1^ of MIP, whilst in milk, ENRO and danofloxacin, whose maximum residue limits have been fixed at 0.28 μM and 0.08 μM, respectively, could be selectively measured and distinguished from other families of antibiotics.

Turner et al. incorporated *N*-2-propenyl-(5-dimethylamino)-1-naphthalene sulphonamide into imprinted polymer films as a fluorescent indicator for the detection of nitroaromatic compounds in the vapor phase [90]. Binding of the explosives was detected within a few min as a quenching of fluorescence. Enhancement of fluorescence upon binding template is less common, but examples exist. Ivanova-Mitseva et al. prepared nanoparticles by grafting to a dendrimer core simultaneously modified with dansyl amide groups and a dialkyldithiocarbamate ester (iniferter) [91]. The nanoparticles produced showed a positive fluorescent response to the presence of the template (acetoguanamine) at nanomolar concentrations (LoD = 3.0 × 10^−8^ M), which was selective over close structural analogues. A similar “light-up” detection for amino acid derivatives has been demonstrated with a urea-based functional monomer designed to interact with the carboxylate anion on the template [92]. The polymer showed enantioselective binding of l-phenylalanine benzyl ester at micromolar concentrations.

**Fig. 3:**
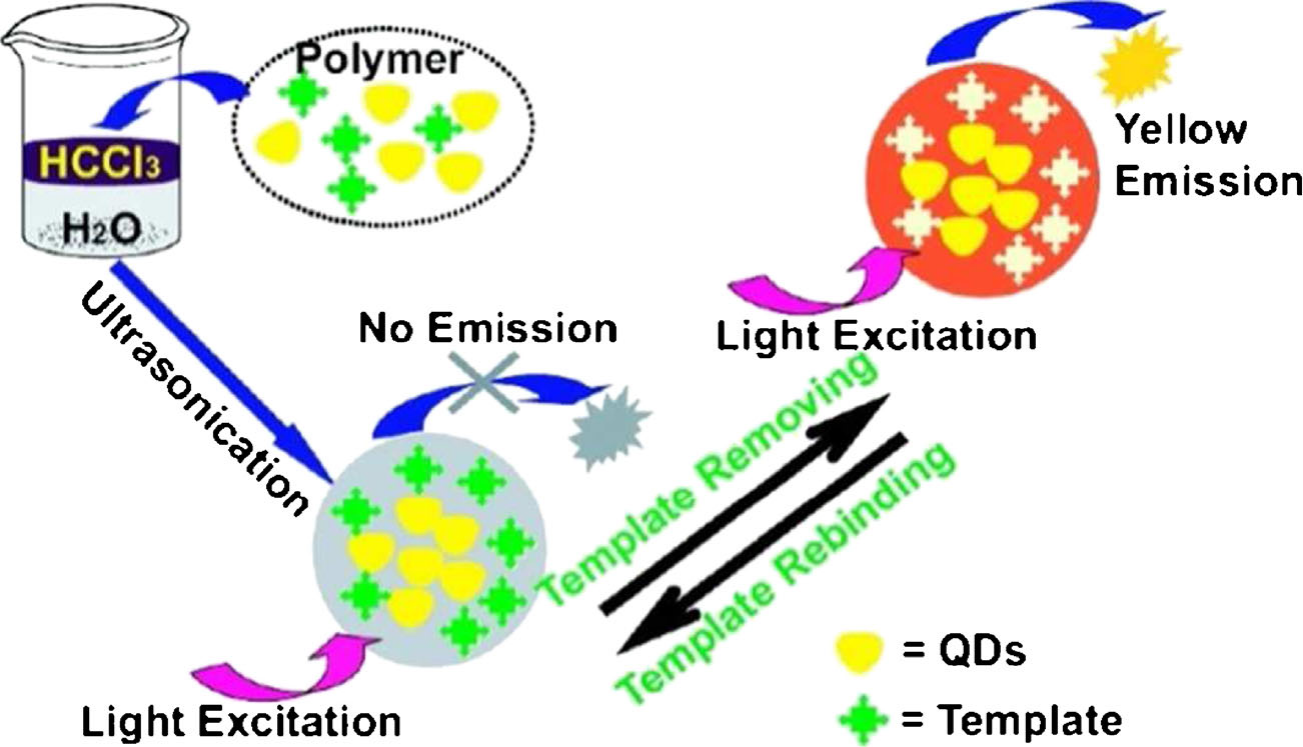
Scheme for the preparation of quantum dots-based molecularly imprinted polymer (QDs-MIP) nanospheres, and the fluorescence quenching effect following rebinding of template as a method of detection [93]. Reprinted with permission from Zhao Y, Ma Y, Li H, Wang L (2012) Analytical Chemistry 84(1):386–395. Copyright 2012 American Chemical Society

An interesting development in homogeneous fluorescence MIAs came as a result of improvements in luminescent nanomaterials. Incorporation of these materials was first demonstrated by Zhao et al., who reported the rational and rapid fabrication of quantum dot (QD)-MIP fluorescent nanospheres capable of recognizing diazinon in aqueous media [93]. Based on energy transfer from the excitation of ZnS:Mn^2+^ (donor) to the absorption of diazinon (acceptor), the fluorescence of the QDs-MIP nanospheres was greatly quenched as the template molecules rebound into the recognition cavities (Fig. 3). The dramatic fluorescence quenching could be applied to the direct and selective fluorescence quantification of diazinon in aqueous media, with the developed assay displaying a linear relationship over the concentration range 50–600 ng mL^−1^. As a proof of concept, the QDs-MIP nanospheres were applied to the analysis of diazinon in tap water samples spiked with 200 ng mL^−1^ of the analyte, with excellent recoveries varying from 98.2 % to 105.4 %, demonstrating the applicability to detection in real environmental water samples without any pretreatment.

**Fig. 4:**
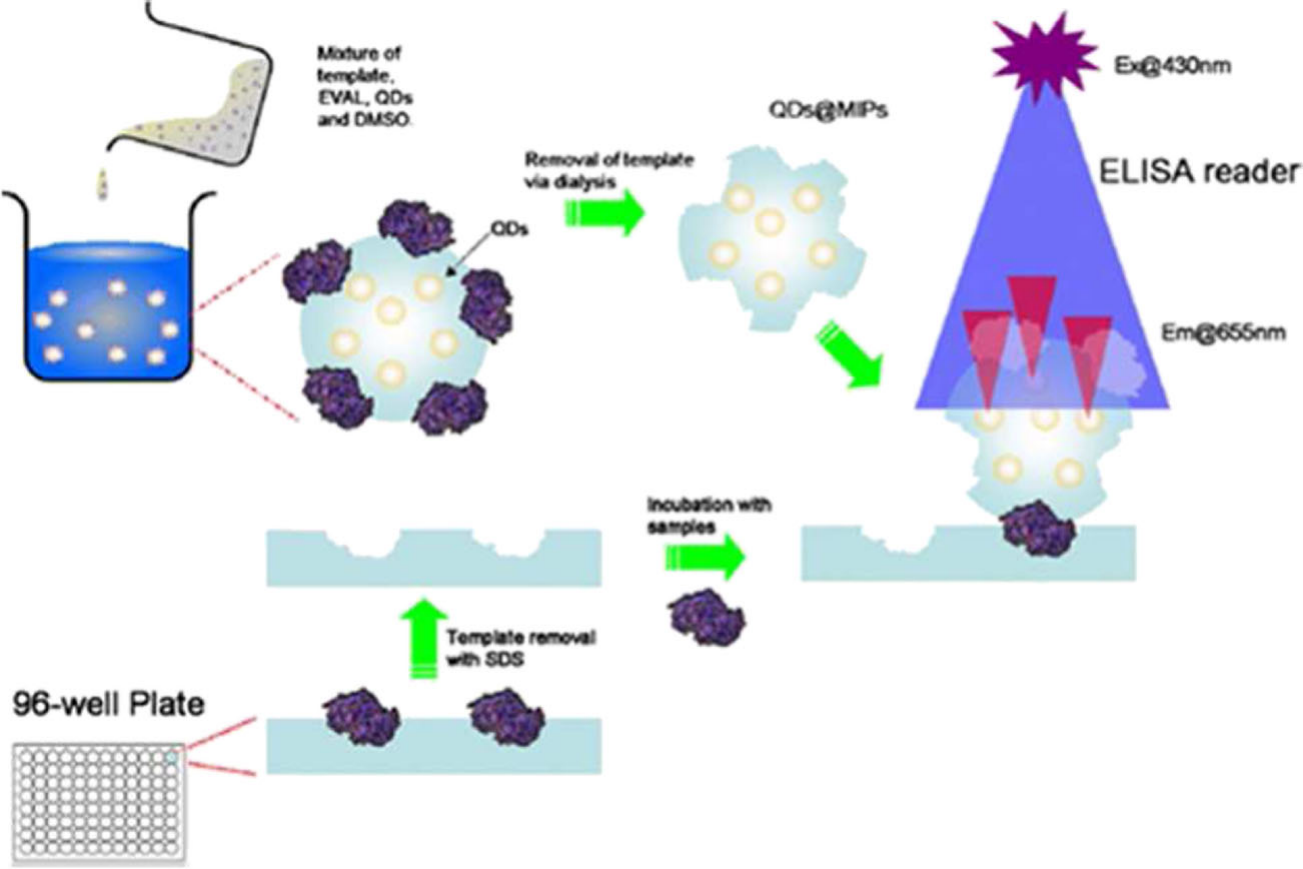
Recognition by template-imprinted poly(ethylene-co-vinyl alcohol)/quantum dots nanoparticles following binding to imprinted polymer coated 96-well microplates to form a sandwich-type assay for protein detection [94]. Reproduced with permission from Springer Science + Business Media: Microchimica Acta. Lee MH, Thomas J, Chen YC, Chin WT, Lin HY (2013) The complete replacement of antibodies by protein-imprinted poly(ethylene-co-vinyl alcohol) in sandwich fluoroimmunoassay. 180:1393–1399, Scheme 1

Incorporation of QDs as a source of fluorescence signaling was also the method of choice adopted by Lee et al. during development of the first MIP sandwich assay [94]. The sandwich fluoroimmunoassay was designed to detect and quantify digestive proteins in saliva, utilizing quantum dots incorporated in protein imprinted poly(ethylene-co-vinyl alcohol) (pEVAL) as a fluorescent signal (Fig. 4). The same polymer (pEVAL) was also used as an imprinted thin film to coat microplate wells as a replacement for primary antibodies in the sandwich assay system. The system relies on the random imprinting of different surface features of the target protein (epitopes) in the primary and secondary polymer components, similar to that obtained with polyclonal antibodies. When applied to measurements of saliva samples, the recovery accuracy attained by this method was ±20 %–25 %, whilst the linear range for amylase, lipase, and lysozyme stock solution were 0.1–10 ng mL^−1^, with the limit of detection as low as 1 pg mL^−1^. These results therefore represented the most sensitive detection yet achieved with MIPs.

### Enzyme-linked MIAs

The use of enzyme-labeling analytes was first implemented as early as 1968, and has since become the most popular method for labeling in immunoassays. This trend has translated over to MIAs also, as traditional problems of incompatibility with water and accessibility of binding sites with the use of enzymes with MIPs have been overcome. Enzyme-labels still suffer the same problems as fluorescent probes with regards to conjugation of the label to the analyte and the effect this consequently has on the recognition and binding of the labeled analogue; however, the commercial availability of many enzymes at low cost and general ease of conjugation offer significant advantages. Additionally, many enzyme labels undergo simple colorimetric/fluorimetric reactions during their application, requiring detection devices no more complex or expensive than a multichannel colorimetric/fluorimetric reader.

The aforementioned difficulty of binding site availability has led to adoption of in situ polymerization of imprinted films on the surface of 96-well plates as the most popular technique for development of biomimetic ELISA-like assays (Table 1). By utilizing a film format, a large surface area can be achieved, whilst control of the film thickness assists in access to binding sites. The method has been used extensively for a variety of templates, with the developed assays being applicable to determination of their respective analytes in environmental water samples [95, 96], soil [96], pork [97], urine [97, 98], vegetables [99], chick feed [100], sea cucumber [101], French fries, and crackers [102].

**Table 1:** Recent examples of MIAs utilising enzyme-labels and in situ prepared imprinted films on the surface of 96-well μL plates

Analyte	Range (μg L^−1^)	IC_50_ (μg L^−1^)	LoD (μg L^−1^)	Ref.
Estrone	0.50–50,000	200 ± 40	8.0 ± 0.2	[92]
Ractopamine	0.01–1000	15.8 ± 3.2	0.01	[97]
Methimazole	0.60–60,000	70.0 ± 4.0	0.9 ± 0.04	[98]
Trichlorfon	3.20–50,000	6800 ± 60	6.8 ± 0.2	[99]
Olaquindox	17.0–50,000	700 ± 60	17 ± 1.6	[100]
Chloramphenicol	0.30–30,000	30.0 ± 2.0	0.9 ± 0.01	[101]
Tribenuron-methyl	0.10–10,000	19.7 ± 1.2	0.3	[96]
Acrylamide	16.0–50,000	8000 ± 0.4	85 ± 4.2	[102]

Recent work reported by Shi et al. describes the development of a MIP-based ELISA for simultaneous multi-pesticide analysis [103]. The chosen template, 4-(dimethoxyphosphorothioylamino)butanoic acid, had been shown to share a common structure and functional groups with organophosphorus pesticides, and so the intention was that this template could be used to produce a MIP with recognition for the organophosphorus class of compounds, rather than just the template. The imprinted film proved to be effective for selectively recognising trichlorfon and acephate, with an *IC*_*50*_ of 12.0 mg L^−1^ and 30.0 mg L^−1^ for each analyte, respectively. Overall, the assay showed linearity from 0.1 to 100,000 μg L^−1^, making it suitable for the desired purpose of determining trace amounts of pesticides in food samples. When subjected to spiked asparagus and cucumber samples, recoveries from 72.1 % to 92.0 % for trichlorfon and 70.0 % to 85.0 % for acephate were achieved.

**Fig. 5:**
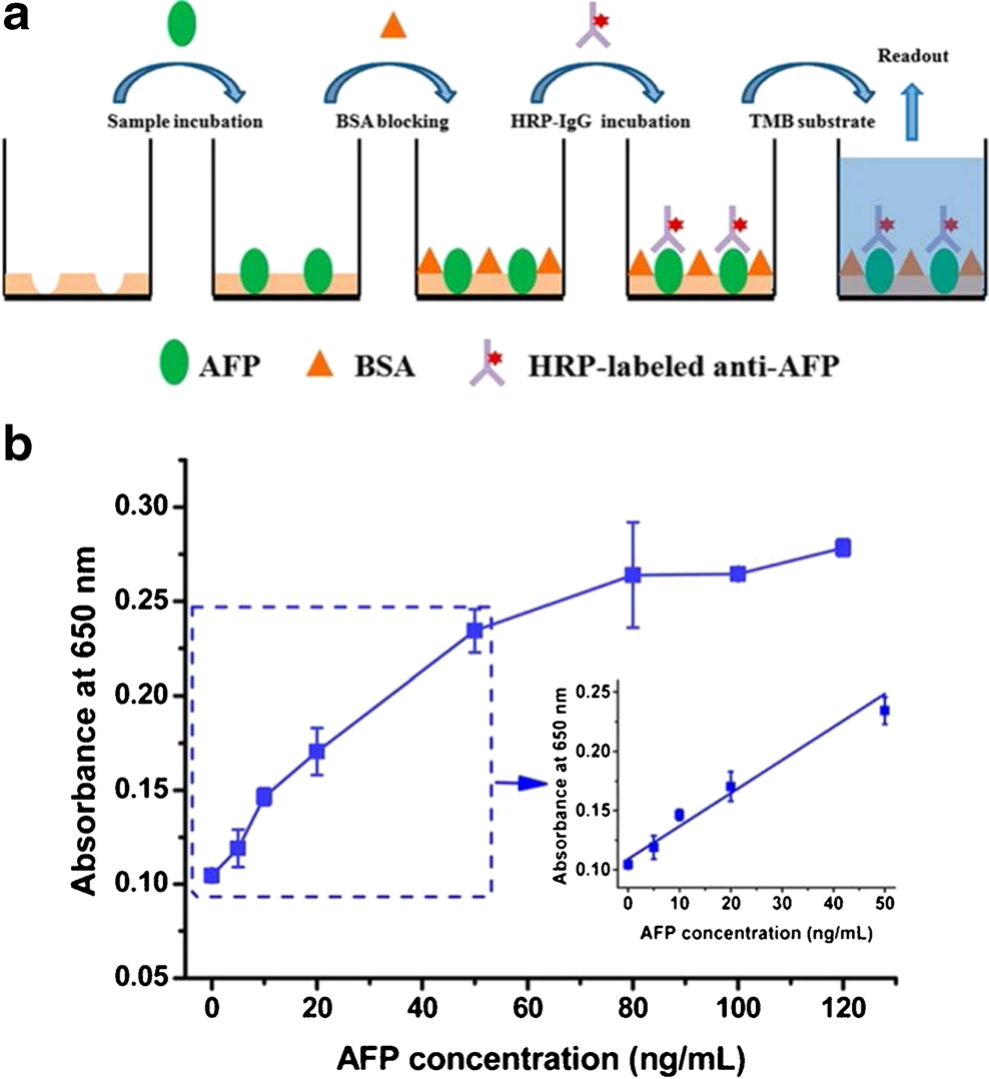
Sandwich ELISA for α-fetoprotein (AFP) following boronate affinity-based oriented surface imprinting [104]. Reprinted with permission from Bi X, Liu Z (2014) Analytical Chemistry 86(1):959–966. Copyright 2014 American Chemical Society

An interesting variation on surface-imprinting was performed in order to achieve the first 96-well microplate MIP ELISA for glycoprotein detection and quantification [104]. In this work, a 96-well microplate was functionalized with a common boronic acid at the well surface, allowing a target glycoprotein to be immobilized by virtue of boronate affinity. Following this, a hydrophilic coating formed by in-water self-copolymerization of aniline was deposited onto the well surface, affording a 3D cavity complementary to the molecular shape of the target following removal with acid (Fig. 5). The group prepared α-fetoprotein (AFP)-imprinted microplates to develop a MIP-based sandwich ELISA, which showed good linearity over the range 0–50 ng mL^−1^. When applied to a human serum sample, the AFP concentration was determined to be 12 ± 2.0 ng mL^−1^, which was in good agreement with the value determined by radioimmunoassay (10 ng mL^−1^), showing a promising prospect of the proposed method in clinical diagnostics.

**Fig. 6:**
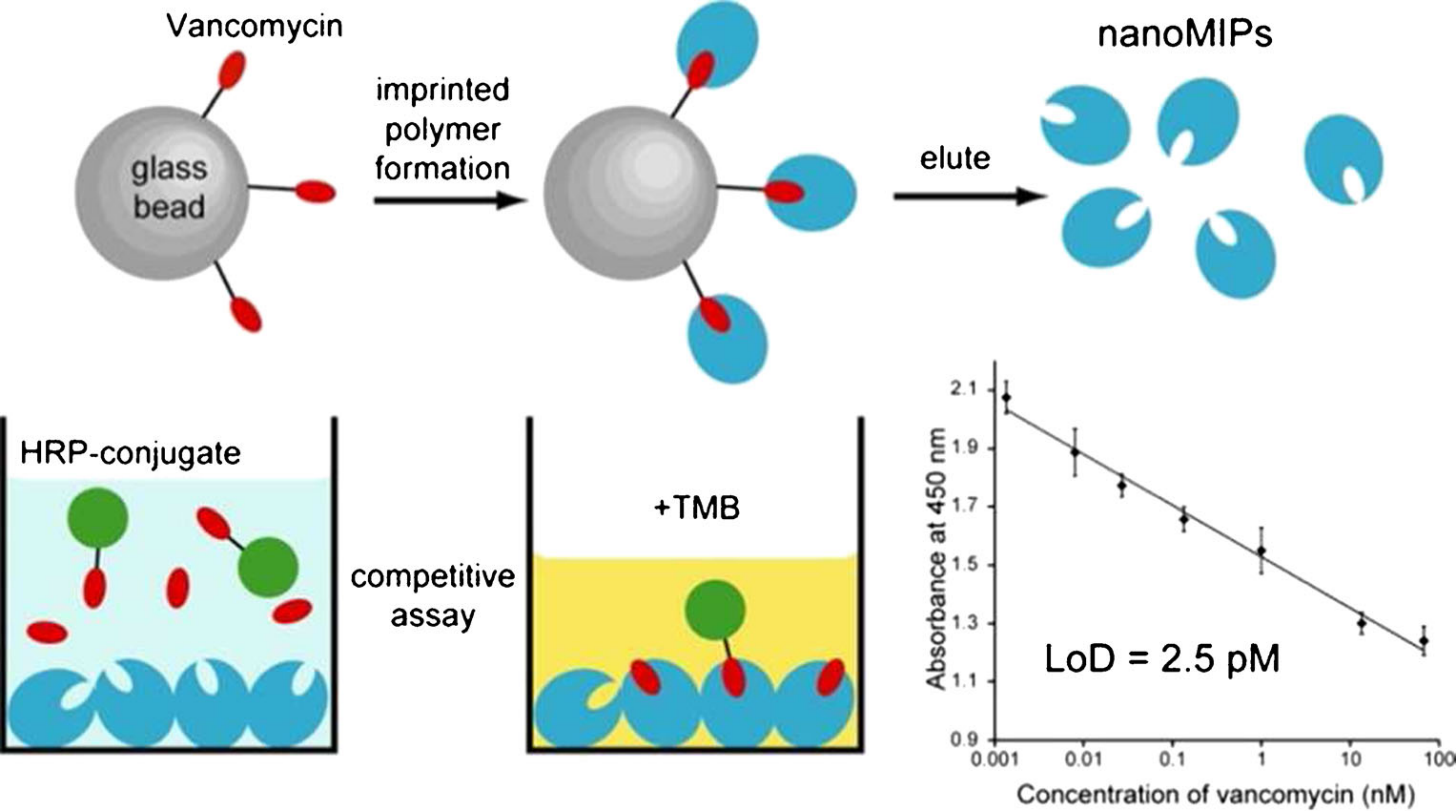
ELISA utilizing nanoMIPs synthesized using a solid phase protocol [106]. Reprinted (adapted) with permission from Chianella I, Guerreiro A, Moczko E, Caygill JS, Piletska EV, De Vargas Sansalvador IMP, Whitcombe MJ, Piletsky SA (2013) Analytical Chemistry 85(17):8462–8468. Copyright 2013 American Chemical Society

Although impressive results have been achieved using molecularly imprinted films, attempts to improve upon this method have been made. With regards to the films used in these assays, their resemblance to polyclonal antibodies gave rise to high levels of nonspecific binding, whilst their manufacture relied on manual, labor intensive methods of synthesis. The assays themselves utilized complex immobilization protocols and lacked generality, requiring substantial modification to the analytical procedures traditionally used in ELISA. In an attempt to resolve some of these problems, Poma et al. developed a method for solid-phase synthesis of MIP nanoparticles with pseudomonoclonal binding properties suitable for automation in a computer-controlled reactor [105]. To demonstrate the potential of materials prepared in this manner, a novel assay for vancomycin directly replacing antibodies with molecularly imprinted polymer nanoparticles in ELISA was proposed [106]. In order to utilize previously synthesized MIP nanoparticles, a simple and straightforward technique for coating microplate wells was required. This was achieved through physical adsorption by allowing a solution of nanoMIPs to evaporate to dryness within each of the microplate wells, removing the necessity for a complex immobilization method or in situ formation of the imprinted material through polymerization in the test wells. Following immobilization, the nanoMIPs could be used in competitive binding experiments between free and HRP-labeled vancomycin (Fig. 6). The assay was capable of measuring vancomycin in buffer and in blood plasma within the range of 0.001–70 nM, a sensitivity three orders of magnitude better than a previously described ELISA based on antibodies. The generic nature of nanoMIP preparation by solid-phase synthesis suggests that assays for many more analytes may also be created in this manner.

### Other MIA formats

Although the majority of molecularly imprinted assays fall into the previously discussed categories, several novel assay types have been developed utilizing the unique properties of MIPs.

**Fig. 7:**
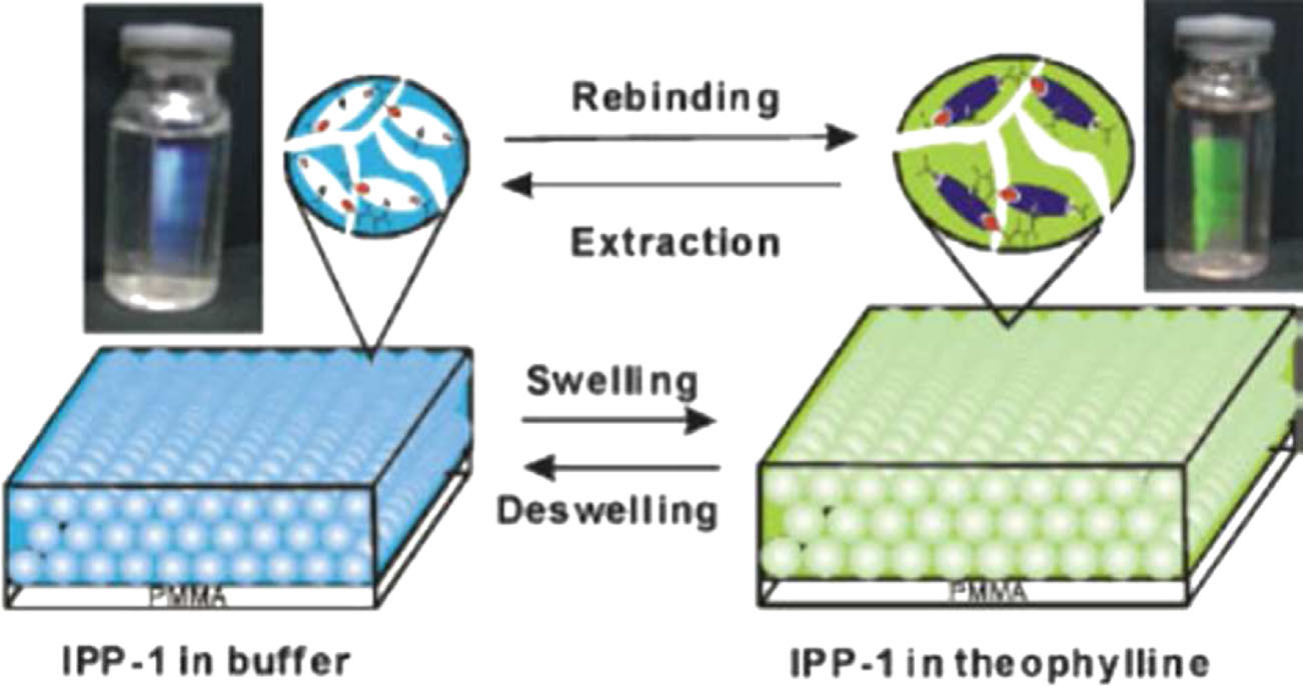
Schematic illustration of the created imprinted photonic polymers (IPP) structure and the color change as a result of swelling/deswelling following rebinding or extraction of analyte [107]. Reproduced with permission from: Ultrasensitive Specific Stimulant Assay Based on Molecularly Imprinted Photonic Hydrogels, Hu XB, Li GT, Li MH, Huang J, Li Y, Gao YB, Zhang YH. Advanced Functional Materials, Vol. 18:4, Copyright © 2008, John Wiley and Sons

**Fig. 8:**
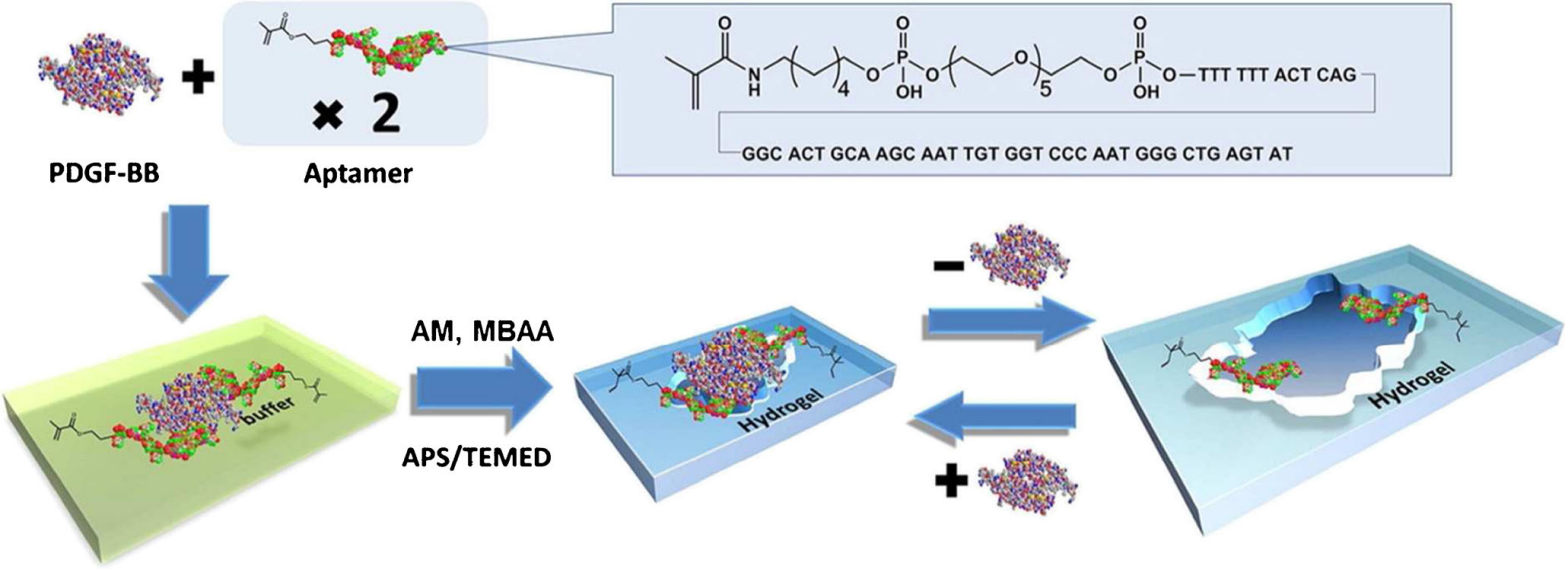
Aptamer-based hydrogels imprinted with thrombin that show macroscopic changes in dimension on binding the target protein down to femtomolar concentration [117]. Reprinted with permission from Bai W, Gariano NA, Spivak DA (2013) Journal of the American Chemical Society 135(18):6977–6984. Copyright 2013 American Chemical Society

Taking advantage of the swelling/deswelling behavior of hydrogels, Hu et al. developed an ultrasensitive specific stimulant assay based on molecularly imprinted photonic hydrogels [107]. In this work, colloidal crystals and molecular imprinting were combined to prepare imprinted photonic polymers (IPP) with three-dimensional, highly-ordered, macroporous structures, which could be used to optically determine analytes by means of the shift of the Bragg diffraction attributable to a change of the periodic lattice spacing. The IPP hydrogels swell in response to chemical stimuli, giving rise to a visually perceptible color change, which can easily be implemented into a rapid and sensitive assay (Fig. 7). IPP-hydrogel films against theophylline and (1R,2S)-(−)-ephedrine both exhibited high sensitivity and selectivity, enabling the quantification of as low as 0.1 fM. concentration of analyte even in a competitive urinous buffer. Similar detection methods have been demonstrated in colloidal crystal and inverse opal configurations. Although many of these have been described as sensors, rather than assays, they are worthy of mention since they operate in the same manner. Analytes determined in this fashion include bisphenol A [108, 109], organophosphorus compounds [110], imidacloprid [111], glucose [112], amino acids [113], progesterone [114], tetracycline [115], and 17β-estradiol [116]. Volume changes have also been employed in the detection of proteins in hydrogels imprinted using novel functional monomers based on aptamers [117]. In this work, the protein thrombin was used as the template with two distinct polymerizable aptamer sequences as functional monomers chosen to bind to different regions of the protein surface. After template removal the hydrogel could be used to detect protein binding by changes in the macroscopic dimensions (shrinkage) of the gel down to femtomolar concentrations (Fig. 8).

Similarly to fluorescence, chemiluminescence has also been employed as a signaling method for MIAs. An assay for dipyridamole has been developed utilizing light emitted from dipyridamole peroxyoxalate chemiluminescence (PO-CL) reaction as a means of detection [118]. MIP microspheres of 0.7 μm diameter were prepared using precipitation polymerization with methacrylic acid as functional monomer and trimethylolpropane trimethacrylate as cross-linker in the presence of dipyridamole, with poly(vinyl alcohol) utilized to immobilize the imprinted polymers to the walls of 96-microtiter well plates. Following sample incubation, the amount of polymer-bound dipyridamole was determined using a high-resolution charge coupled device camera to measure the light emitted from the PO-CL reaction. Under optimal conditions, the relative chemiluminescence imaging intensity was proportional to dipyridamole concentration from 0.02 to 10 μg mL^−1^, with the assay format able to perform 96 independent measurements simultaneously in 30 min.

**Fig. 9:**
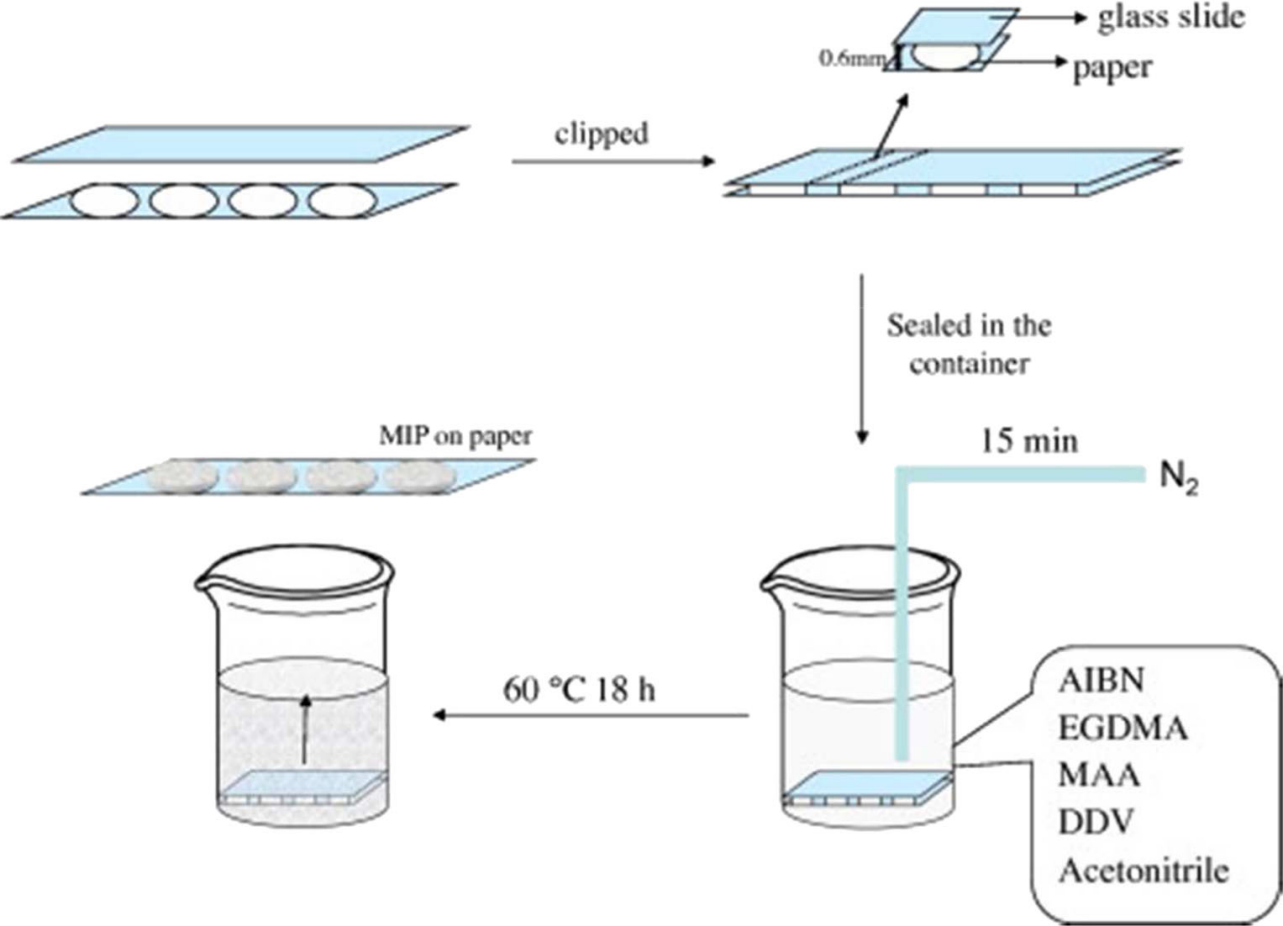
The procedure used to prepare MIP on paper [119]. Reprinted from Spectrochimica Acta Part A: Molecular and Biomolecular Spectroscopy, Liu W, Guo YM, Luo J, Kou J, Zheng HY, Li BX, Zhang ZJ. A molecularly imprinted polymer based a lab-on-paper chemiluminescence device for the detection of dichlorvos, 141:51–57. Copyright 2015, with permission from Elsevier

A molecularly imprinted polymer-based lab-on-paper chemiluminescence device for the detection of dichlorvos (DDV) was reported by Liu et al., generating chemiluminescence signals following reaction of DDV, luminol, and H_2_O_2_ in alkaline medium, allowing for a powerful and sensitive tool for selective monitoring of DDV [119]. The MIP layer was adsorbed onto the paper surface, whilst the depth was controlled at 600 μm by stacking glass slides with double-sided tape of 600 μm depth (Fig. 9). When applied to vegetable samples, the device was effective from 3.0 ng mL^−1^ to 1.0 μg mL^−1^ with a detection limit of 0.8 ng mL^−1^. Whilst the work demonstrates the promise of chemiluminescence-based detection for paper microfluidic chips, the adaptability of this device to the analysis of other analytes could be limited, as they, like DDV, would be required to elicit a chemiluminescence signal following addition of luminol/H_2_O_2_.

**Fig. 10:**
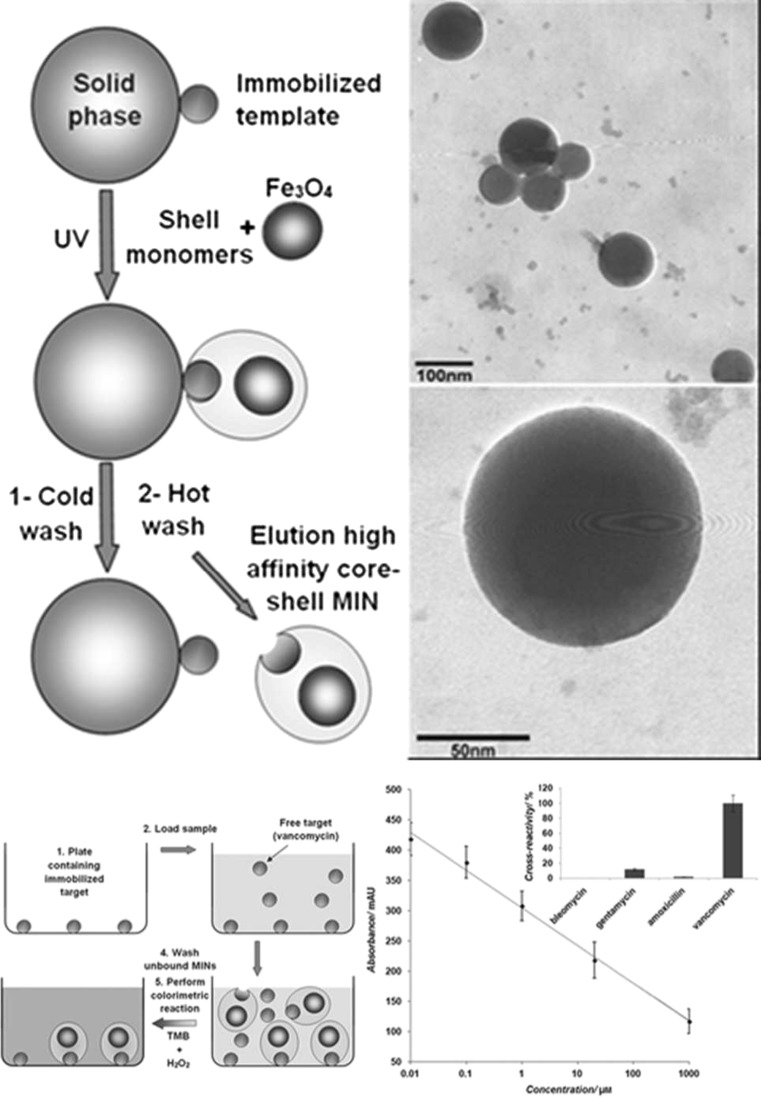
Schematic of the solid-phase synthesis protocol with addition of Fe_3_O_4_ for preparation of peroxidase-mimicking core-shell MIN (top left) and TEM image of the obtained Fe_3_O_4_-MIN particles (top right). The assay format (bottom left) and calibration curve (bottom right) are also shown [120]. Reproduced with permission from: Introducing MINA - The Molecularly Imprinted Nanoparticle Assay. Shutov RV, Guerreiro A, Moczko E, de Vargas-Sansalvador IP, Chianella I, Whitcombe MJ, Piletsky SA, Small, Vol. 10:6. Copyright © 2014 John Wiley and Sons

Although the replacement of antibodies with synthetic mimics has been the focus of biomimetic ELISA-like assays, the added advantages (mainly storage/thermal stability and low cost) afforded by use of these materials is not effectively exploited if the assay system still requires the use of a biological reporter enzyme. In an attempt to rectify this, Shutov et al. reported the integration of catalytically active Fe_3_O_4_ with molecularly imprinted nanoparticles (MINs) as combined recognition and signaling functionalities in a core-shell nanoparticle format to develop the first ELISA-like assay (MINA) to completely replace all biologics with synthetic analogues [120]. The intrinsic peroxidase mimicking activity of Fe_3_O_4_ nanoparticles makes them attractive substitutes for enzymes in a variety of assays, with suitable catalytic activity over a broad range of temperatures, low cost/long shelf life, and ease of manufacture. A variation of the solid-phase imprinting protocol was utilized to produce the composite core-shell Fe_3_O_4_-MIN, using vancomycin as template (Fig. 10). Subsequent magnetic separation ensured that only high-affinity nanoparticles containing the catalytic Fe_3_O4 core were recovered from the process. By immobilizing the template (vancomycin) to the surface of well plates, a competitive assay could be performed using the previously synthesized core-shell nanoparticles, with quantification made possible through the oxidation of 3,3′,5,5′-tetramethylbenzidine (TMB) to give a colorimetric response proportional to the quantity of Fe_3_O_4_ catalyst bound to the template. The developed assay was effective over the range of 10 nM to 1 mM, retaining applicability even in complex sample matrices such as porcine serum, although this did require use of a spacer between immobilized vancomycin and the well surface.

**Fig. 11:**
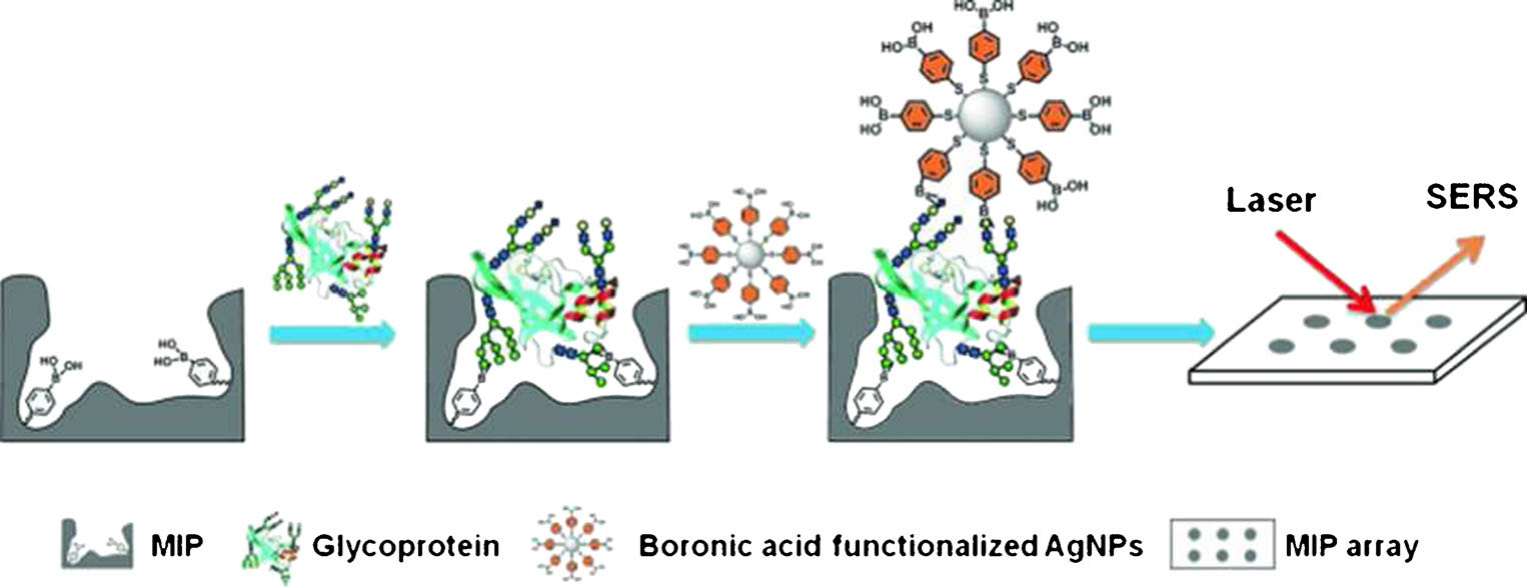
Schematic representation of the boronate-affinity sandwich assay of glycoproteins [121]. Reproduced with permission from: A Boronate Affinity Sandwich Assay: An Appealing Alternative to Immunoassays for the Determination of Glycoproteins, Ye J, Chen Y, Liu Z (2014) Angewandte Chemie International Edition, Vol. 53:39. Copyright © 2014, John Wiley and Sons

A number of sandwich-type assays have been developed, achieving incredible sensitivity surpassing that of other previously mentioned methods. A new approach, termed the boronate-affinity sandwich assay (BASA), was applied for the specific and sensitive determination of trace glycoproteins in complex samples [121]. The technique relies on the formation of sandwiches between boronate-affinity molecularly imprinted polymers, target glycoproteins, and boronate-affinity surface-enhanced Raman scattering (SERS) probes (Fig. 11). In this way, the MIP ensures the specificity, whilst the SERS detection provides sensitivity. The feasibility of the BASA approach for real-world applications was demonstrated by an assay of the glycoprotein α-fetoprotein in human serum. The MIP array exhibited a linear response toward AFP within the range of 1 ng mL^−1^ to 10 μg mL^−1^, and was able to determine the analyte concentration in good agreement with results from other methods (13.8 ± 3.3 ng mL^−1^ compared with 12.0 ± 2.0 ng mL^−1^).

**Fig. 12:**
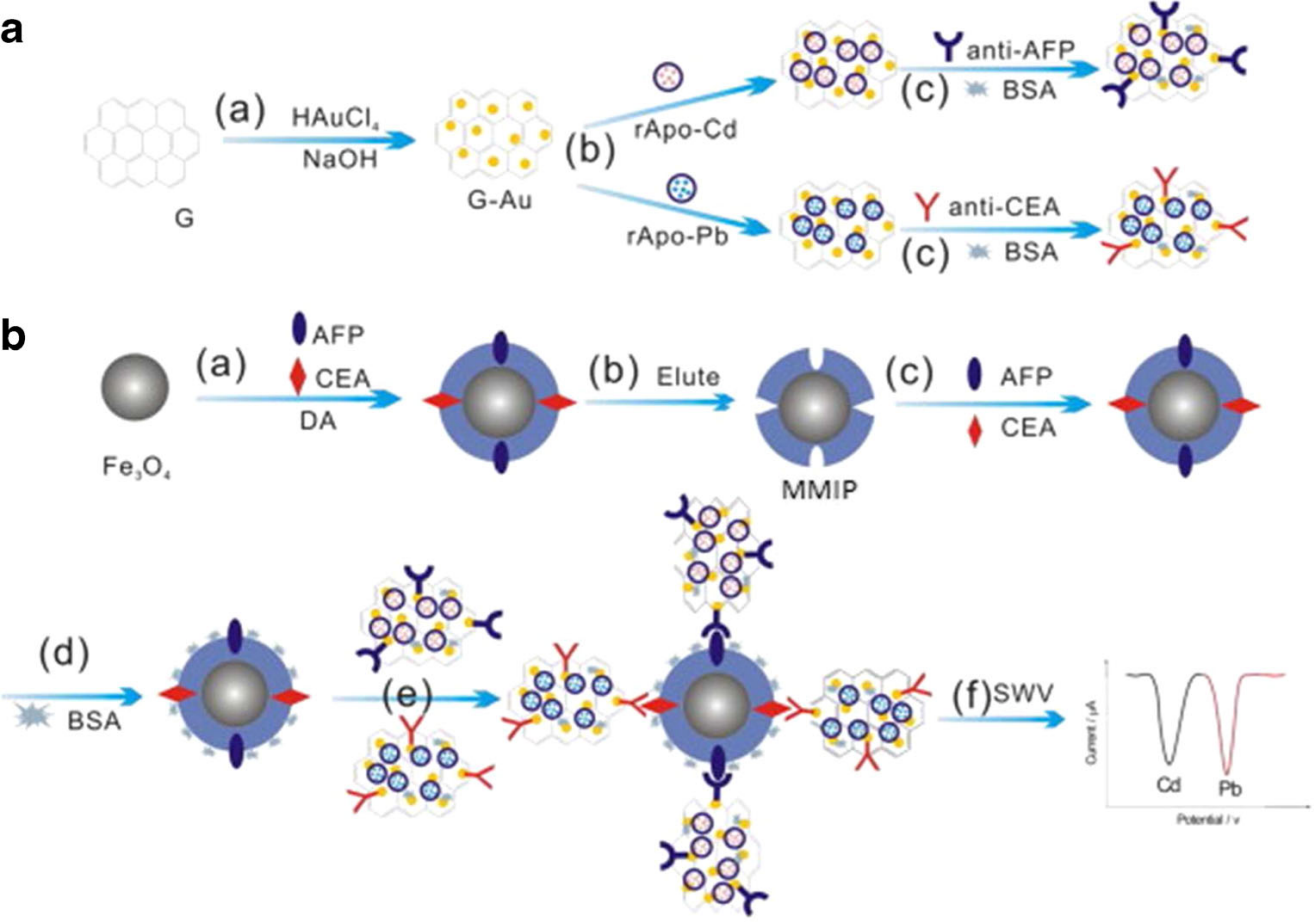
Schematic representation of simultaneous electrochemical immunoassay. **(A)** preparation of signal tags: **(a)** in situ reducing HAuCl_4_ onto graphene (G) to form G–Au; **(b)** immobilization of labels (rApo-M); **(c)** labeling with anti-AFP and anti-CEA and blocking of excess active sites with BSA (1.0 wt% ). **(B)** synthesis of the capture probes and electrochemical detection: **(a)** polymerization of DA to form a PDA coating on Fe_3_O_4_ in the presence of template proteins; **(b)** Eluting with SDS to remove embedded template proteins and obtain MMIP; **(c)** recognition with targets analytes (AFP and CEA); **(d)** blocking with BSA; **(e)** antigen–antibody specific reaction with above signal tags; **(f)** magnetic separation and electrochemical detecting with SWV [122]. Reprinted from Biosensors and Bioelectronics. Wang D, Gan N, Zhang HR, Li TH, Qiao L, Cao YT, Su XR, Jiang S. Simultaneous electrochemical immunoassay using graphene–Au grafted recombinant apoferritin-encoded metallic labels as signal tags and dual-template magnetic molecular imprinted polymer as capture probes, 65:78–82. Copyright 2015, with permission from Elsevier

A further novel sandwich-type immunoassay for simultaneous determination of AFP and carcinoembryonic antigen (CEA) using graphene–Au grafted recombinant apoferritin-encoded metallic labels (rApo-M) loaded with Cd and Pb ions with dual-template magnetic MIPs (MMIPs) as capture probes was designed by Wang et al. [122]. After a sandwich-type immunoreaction, the labels were captured at the surface of MMIPs, allowing electrochemical stripping analysis of the metal components from the immunocomplex to provide a means of quantification based on the peak currents of Cd and Pb (Fig. 12). Experimental results showed that the assay could simultaneously detect AFP and CEA in a single run with a dynamic range of 0.001–5 ng mL^−1^. The possibility to expand the number of analytes for simultaneous analysis by implementing more rApo nanoparticles (including Pb, Cd, Cu, and Zn) as distinguishable labels shows promising potential for this approach in clinical detection of multianalytes.

**Fig. 13:**
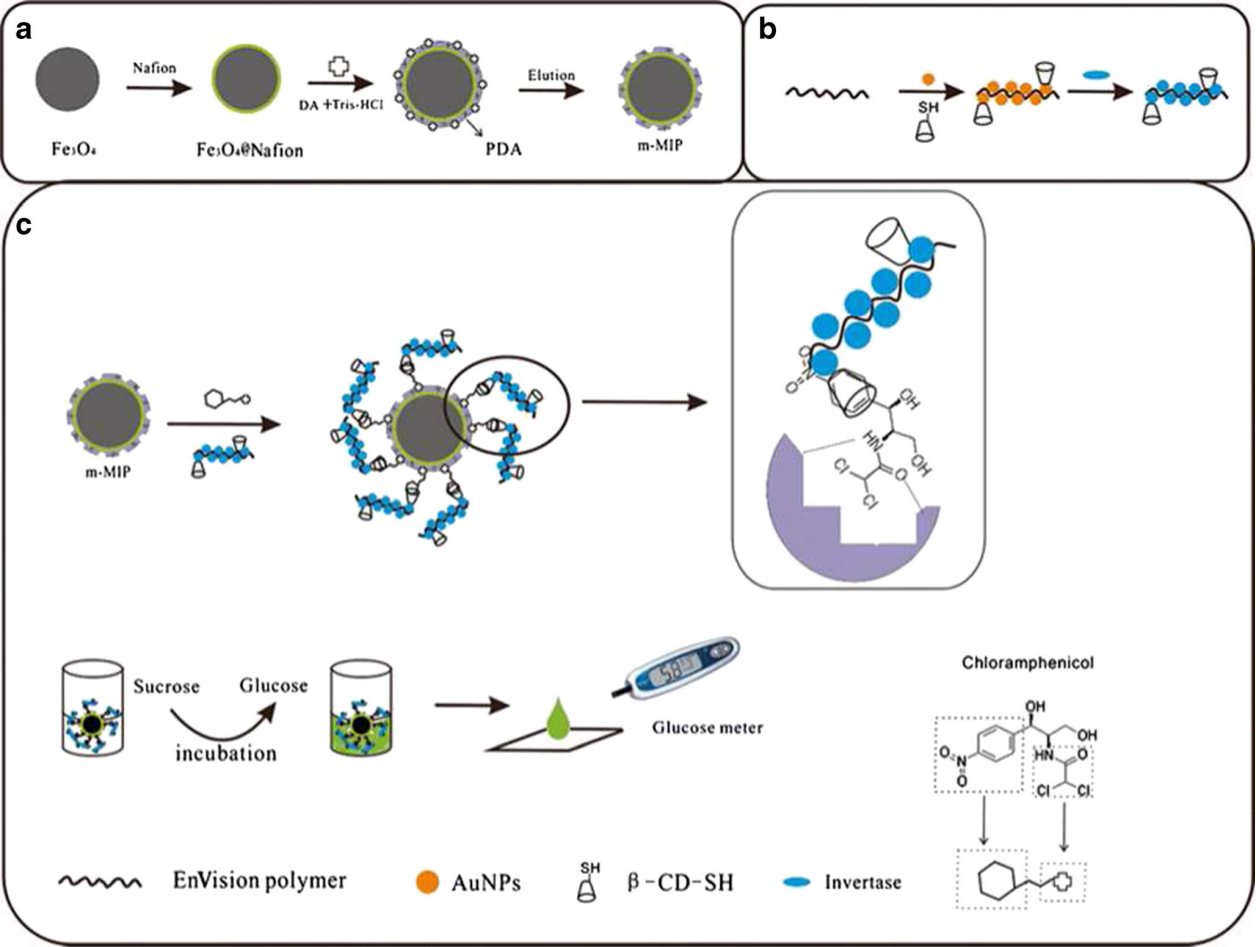
**a** Preparation of magnetic molecularly imprinted polymers. **b** EnVision reagent (EV)-Au-β-cyclodextrin/invertase signal tag preparation. **c** Scheme for the sandwich assay [123]. With permission from Springer Science + Business Media: Analytical and Bioanalytical Chemistry. A portable and antibody-free sandwich assay for determination of chloramphenicol in food based on a personal glucose meter, 407:2499–2507. Chen S, Gan N, Zhang HR, Hu FT, Li TH, Cui H, Cao YT, Jiang QL (2015) Fig. 1. Copyright 2015, with permission from Elsevier

By taking advantage of the inherent chemical properties of chloramphenicol (CAP), a portable and antibody-free sandwich assay for determination of chloramphenicol in food based on a personal glucose meter was developed by Chen et al. [123]. The assay utilized polydopamine molecularly imprinted film modified Fe_3_O_4_ nanoparticles and a β-cyclodextrin (β-CD)/invertase bioconjugate for recognition and subsequent glucose generation. A fragment imprinting technique was adopted for the synthesis of the polymer film, in which 2,2-dichloroacetamide was used as template. This enabled affinity for a section of CAP resembling the used template, without interfering with the nitrophenol fragment in CAP. β-Cyclodextrin is known to combine with nitrophenol to form a host–guest complex by means of the hydrophobic cavity, and so this exposed region following MIP binding could be utilized for attachment of a β-CD-based signal tag to form a sandwich-type complex for CAP detection. Invertase was selected for conjugation to β-CD, where it could facilitate the generation of glucose from sucrose to elicit a measurable response using a personal glucose meter (Fig. 13). Using this method, the concentration of CAP was found to be proportional to the amount of glucose formed, which could qualitatively assess the CAP with a dynamic range of 0.5–50 ng mL^−1^ and a detection limit of 0.16 ng mL^−1^. Although an elegant strategy, there is great dependence on the structure of the analyte for this method to be applicable because of the need for a nitrophenol moiety to facilitate β-CD complexation, and so the number of substrates able to be analyzed in this manner is limited.

Binding of analytes to the specific recognition sites of imprinted polymers results in a change in the heat-transfer resistance of the materials, which can be used as a sensing or assay technique for their detection. The method (heat-transfer method) has been used as a means of quantifying a range of analytes, including l-nicotine, histamine, and serotonin [124, 125] and mammalian cells, including cancer cells [125–128]. The method is sensitive, does not require labels, and is compatible with biological entities.

## Prospects for diagnostic applications

The motivation for developing assays employing MIPs in place of antibodies has been the advantages that these materials would bring to the field. Generally, MIP development is shorter and less expensive than antibody development, targets do not require conjugation to immunogenic proteins, experimental animals are not involved in the process, and MIPs do not require cold storage and cold-chain logistics. Barriers to adoption of these new technologies may be uncertainty over security of supply and the perception that changes need to be made in manufacturing practices and plant in order to make the switch from antibodies to MIPs. This need not be the case, however, as several groups have demonstrated assays with nanoMIPs that have been used as direct replacements for antibodies in a number of assay formats.

In the medical diagnostics area, there is always concern about the possibility of false positives and false negatives in any diagnostic test. The former can lead to misdiagnosis and inappropriate treatment and the latter to the failure to diagnose a potentially life-threatening condition. It should not be forgotten, however, that these concerns also apply to antibody-based tests and, indeed, to any technology used in diagnosis based on molecular recognition. The implication is that new methods should be validated against existing tests and analytical procedures that use more robust methods (such as LC-MS-MS) that provide unambiguous identification and quantitation of the analyte. More studies in this vein would certainly support the case for the adoption of MIPs by the diagnostic industry.

Many biomarkers for disease diagnosis and monitoring are peptides and proteins. Reports of protein imprinting in the literature have greatly increased in recent years; however, proteins are difficult templates to work with and not all reports provide strong evidence for imprinting. Kryscio et al. have shown that the structure of proteins typically employed as templates are adversely affected by exposure to monomers and cross-linkers commonly used in imprinting [129, 130]. Verheyen and co-workers have also highlighted the problems of nonspecific interactions with polymers carrying charged monomers, which can overwhelm specific binding to MIPs; they also point out the dangers of template removal using SDS and acetic acid, which has led to a number of misleading results [131]. They argue that high binding affinity for proteins can only arise with a combination of hydrogen-bonding, electrostatic and hydrophobic interactions in the correct balance. These and other issues have been raised in other reviews [132, 133], which recommend that surface imprinting approaches be employed with whole protein templates to avoid entrapment and poor binding kinetics. They also point out that “epitope” imprinting [134] avoids many of the pitfalls associated with imprinting macromolecules, as long as the nonspecific binding issue is addressed.

## Conclusions and future prospects

During the last decade, significant progress has been made with regards to molecularly imprinted sorbent assays. Many of the problems that inhibited the growth of the area have been resolved following improvements in synthetic methods and a greater understanding of the molecular imprinting process, with fluorescent and enzyme-linked MIAs now commonplace. Recent years have seen a move away from traditional “bulk” MIP synthesis in favor of particle-based syntheses; in particular, MIP nanoparticles hold great promise as they are more easily incorporated into existing assays formats. Composite architectures with other nanomaterials (such as quantum dots, gold nanoparticles, carbon nanotubes, and graphene) may provide novel detection mechanisms and higher sensitivity. MIPs grafted to the surface of microplate wells hold promise but are unlikely to be adopted by industry because of manufacturing difficulties. Of particular interest are homogeneous assays that do not require separation steps since they simplify analysis and reduce the possibility of errors in measurement. In terms of read-out, colorimetric methods require the simplest instrumentation, which could be a hand-held colorimeter or, in some cases, a simple color chart might suffice. Other read-out methods such as fluorescence detection may require more sophisticated or expensive instruments suitable for the general practitioner’s office or hospital laboratory. None of these considerations differ very much from antibody-based tests, and the storage requirements for MIPs are less demanding.

Challenges still remain to be overcome; the lack of generality amongst assay formats and development is a discouraging factor against the adoption of MIAs over conventional immunoassays. The dawn of automated MIP-nanoparticle synthesizers and solid-phase selection processes to isolate only high-quality MIPs shows great promise for the development of a universal strategy for assay generation, if these technologies manage to live up to their potential.

Interest continues to grow in the field of molecular imprinting technology as evidenced by the constantly expanding quantity of literature on the subject, indicating a bright future for the development of molecularly imprinted sorbent assays.
